# Interobserver variability of 3.0-tesla and 1.5-tesla magnetic resonance imaging/computed tomography fusion image–based post-implant dosimetry of prostate brachytherapy

**DOI:** 10.1093/jrr/rrz012

**Published:** 2019-05-13

**Authors:** Kenta Watanabe, Norihisa Katayama, Kuniaki Katsui, Toshi Matsushita, Atsushi Takamoto, Hiroki Ihara, Yasutomo Nasu, Mitsuhiro Takemoto, Masahiro Kuroda, Susumu Kanazawa

**Affiliations:** 1 Department of Radiology, Okayama University Medical School, 2-5-1 Shikata-cho, Kitaku, Okayama, Japan; 2 Department of Proton Beam Therapy, Okayama University Medical School, 2-5-1 Shikata-cho, Kitaku, Okayama, Japan; 3 Department of Radiology Medical Support Division Okayama University Hospital, 2-5-1 Shikata-cho, Kitaku, Okayama, Japan; 4 Department of Urology, Okayama University Medical School, 2-5-1 Shikata-cho, Kitaku, Okayama, Japan; 5 Department of Radiation Oncology, Japanese Red Cross Society Himeji Hospital, 1-12-1 Shimoteno, Himeji, Hyogo, Japan; 6 Radiological Technology, Okayama University Graduate School of Health Sciences, 2-5-1 Shikata-cho, Kitaku, Okayama, Japan

**Keywords:** prostate brachytherapy, contouring variability, magnetic resonance imaging, computed tomography, MRI/CT fusion, post-implant dosimetry

## Abstract

This study aimed to compare the interobserver variabilities in magnetic resonance imaging (MRI)/computed tomography (CT) fusion image–based post-implant dosimetry of permanent prostate brachytherapy (PPB) between 1.5-T and 3.0-T MRI. The study included 60 patients. Of these patients, 30 underwent 1.5-T MRI and CT 30 days after seed implantation (1.5-T group), and 30 underwent 3.0-T MRI and CT 30 days after seed implantation (3.0-T group). All patients received PPB alone. Two radiation oncologists performed MRI/CT fusion image–based post-implant dosimetry, and the interobserver variabilities of dose–volume histogram (DVH) parameters [dose (Gy) received by 90% of the prostate volume (prostate D90)], percentage of the prostate volume receiving at least the full prescribed dose (prostate V100), percentage of the prostate volume receiving at least 150% of the prescribed dose (prostate V150), dose (Gy) received by 5% of the urethral volume (urethral D5) and the urethral volume receiving at least 150% of the prescribed dose (urethral V150)] were retrospectively estimated using the paired Student’s *t* test and Pearson’s correlation coefficient. The Pearson’s correlation coefficients of all DVH parameters were higher in the 3.0-T group than in the 1.5-T group (1.5-T vs 3.0-T: prostate D90, 0.65 vs 0.93; prostate V100, 0.62 vs 0.82; prostate V150, 0.97 vs 0.98; urethral D5, 0.92 vs 0.93; and urethral V150, 0.88 vs 0.93). In the paired Student’s *t* test, no significant differences were observed in any of the DVH parame*t*ers between the two radiation oncologists in the 3.0-T group (0.068 ≤ *P* ≤ 0.842); however, significant differences were observed in prostate D90 (*P* = 0.004), prostate V100 (*P* = 0.011) and prostate V150 (*P* = 0.002) between the oncologists in the 1.5-T group. The interobserver variability of DVH parameters in the MRI/CT fusion image–based post-implant dosimetry analysis of brachytherapy was lower with 3.0-T MRI than with 1.5-T MRI.

## INTRODUCTION

Prostate cancer is one of the most common tumors in men, and permanent prostate brachytherapy (PPB) is one of the definitive treatment options for clinically localized prostate cancer [1]. PPB has been used globally since the 1990s because of its short treatment period, excellent disease control rate, acceptable toxicity profile and low cost [2–5].

Post-implant dosimetry analysis of permanently implanted seeds in the prostate provides an estimate of the dose distribution delivered to the patient according to measured seed positions rather than planned seed positions [6]. Additionally, post-implant dosimetry analysis is an important component of prostate brachytherapy programs as it helps predict patient prognosis and toxicity and provides valuable feedback to brachytherapists [7]. Thus, post-implant dosimetry analysis should be performed in all patients [8].

For a meaningful post-implant dosimetry analysis, it is crucial to delineate a patient’s structural contours and determine source locations accurately [6]. As magnetic resonance imaging (MRI) has excellent soft-tissue contrast and computed tomography (CT) allows automatic seed localization, it is favorable to use MRI/CT fusion images in post-implant dosimetry analysis [9]. The American Brachytherapy Society guidelines published in 2012 recommend the use of MRI/CT fusion images for post-implant dosimetry analysis to improve the reproducibility of contouring and the reliability of dosimetry [10–12]; however, uncertainties in MRI/CT fusion–based data could lead to a greater variability in the dose (Gy) received by 90% of the prostate volume (prostate D90) [13]. Because the MRI/CT fusion procedure is performed by seed matching in most cases, the identification of the seeds used for prostate brachytherapy on MRI remains difficult [14].

Conventionally, 1.5-tesla (T) MRI has been used to obtain fusion images; however, 3.0-T MRI is being increasingly used recently owing to the spread of 3.0-T MRI equipment [9, 15]. Identification of the prostate anatomical structure and identification of seeds have been shown to be better with 3.0-T MRI than with 1.5-T MRI, because a higher magnetic field strength increases the signal-to-noise ratio, allowing increased spatial resolution [16]. Thus, 3.0-T MRI might be preferred in the MRI/CT fusion image–based post-implant dosimetry analysis of PPB. However, its reliability has not been appropriately investigated. Only one report has compared the interobserver variability in the contoured prostate volume between 1.5-T and 3.0-T MRI [7]. No study has compared the interobserver variabilities of dose–volume histogram (DVH) parameters in MRI/CT fusion image–based post-implant dosimetry between 1.5-T and 3.0-T MRI. Thus, the present study aimed to compare the interobserver variabilities in DVH parameters for MRI/CT fusion image–based post-implant dosimetry of PPB between 1.5-T and 3.0-T MRI and to determine whether interobserver variability is lower with the 3.0-T MRI approach than with the 1.5-T MRI approach.

## MATERIALS AND METHODS

### Patient population

A total of 60 consecutive patients with low-risk or intermediate-risk prostate cancer (prostate-specific antigen level ≤20 ng/ml; Gleason score 6–7; Union Internationale Contre le Cancer 2009 clinical stage T1–T2) were treated with brachytherapy at Okayama University Hospital between July 2009 and November 2011. All patients were treated with 144 Gy of PPB alone. Of the 60 consecutive patients, 30 underwent 1.5-T MRI and CT 30 days after seed implantation between July 2009 and December 2010 (1.5-T group), and 30 underwent 3.0-T MRI and CT 30 days after seed implantation between December 2010 and November 2011 (3.0-T group).

All patients were treated using loose ^125^I radioactive seeds (Oncoseed; Nihon Mediphysics Co., Tokyo, Japan) loaded with a Mick applicator (Mick Radio-Nuclear Instruments Inc., Bronx, NY, USA). VariSeed ver. 8.0.2 software (Varian Medical Systems Inc., Palo Alto, CA, USA) was used for planning and calculating the final dosimetry.

Permanent implantation involving 0.35 mCi ^125^I seeds was performed under transrectal ultrasound and fluoroscopic guidance. Dose–volume constraints were as follows: percentage of the prostate volume receiving at least the full prescribed dose (prostate V100) > 99%, dose (Gy) received by 1% of the urethral volume (urethral D1) < 220 Gy, and dose (Gy) received by 1% of the rectal volume (rectal D1) <144 Gy.

### Imaging

**Figure 1.**
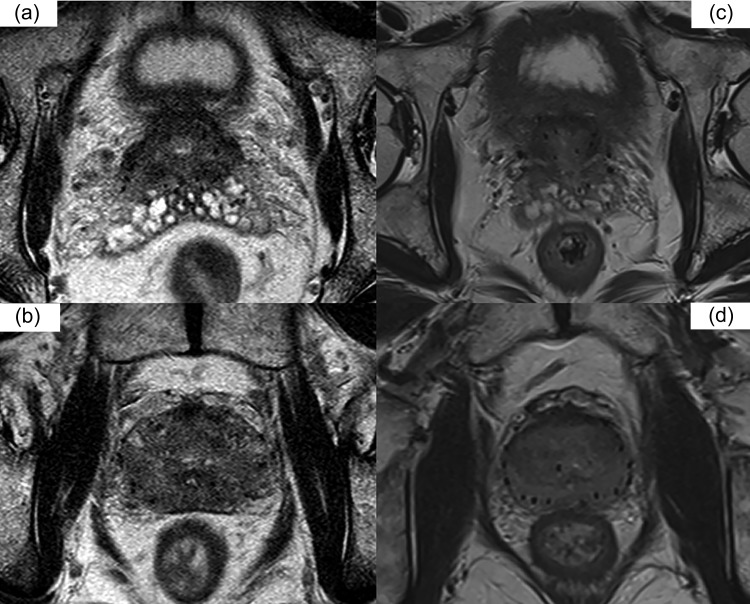
Comparison of the images obtained with 1.5-T magnetic resonance imaging (MRI) and 3.0-T MRI systems. (a, b) 1.5 tesla MRI, (c, d) 3.0 tesla MRI.

The 1.5-T MRI scans were performed (with the patient lying supine) within 15 min after CT, using an Achieva 1.5-T system (Philips Medical Systems, Best, The Netherlands) with a 5-channel SENSE cardiac coil. The pulse sequence involved T2-weighted imaging, and the technical parameters were as follows: echo time (TE)/repetition time (TR), 83/3000 ms; a field of view (FOV), 14 cm; slice thickness, 3 mm without a gap; matrix size, 179 × 256; slice number, 25; flip angle, 170 degrees. Additionally, 3.0-T MRI scans were performed using a Magnetom Verio 3.0-T system (Siemens AG, Erlangen, Germany) with a 16-channel body array anterior and posterior coil. The pulse sequence involved T2-weighted imaging, and the technical parameters were as follows: TE/TR, 99/4000 ms; FOV, 20 cm; slice thickness, 3 mm without a gap; matrix size, 224 × 320; slice number, 32; flip angle, 150 degrees (Fig. 1).

CT scans were performed with the same patient set-up as for MRI, using a CT scanner having 16 detector arrays (Aquilion 64; Toshiba Medical Systems Co., Tokyo, Japan) with 1-mm slice thickness and 1-mm slice interval and a 128-channel, dual-source multi-detector-row CT scanner (SOMATOM Definition Flash; Siemens AG) with 0.6-mm slice thickness and 0.6-mm slice interval. No intravenous contrast was used in the CT or MRI scans, and no urinary catheter was inserted.

Chest radiography (anteroposterior view), kidney–urinary bladder (KUB) radiography and pelvic radiography were performed to check for seed migration on the same day as the MRI and CT scans.

### MRI/CT fusion image–based dosimetry

All images were transferred to the VariSeed software. In CT images, the seeds were detected automatically with the VariSeed software. CT images and seed coordinates that had been detected by Observer 1 were identified and used for the dosimetry of Observer 2. We used the data of post-implant dosimetry performed by Observer 1 ~1 month after seed implantation in clinical practice. Observer 2 had performed post-implant dosimetry of 1.5-T and 3.0-T groups alternately for 2 months. In order to eliminate bias, Observer 2 did not confirm the results of Observer 1. CT and MRI images were electronically fused using the manual-fusion procedure of the VariSeed software. Each observer identified six or more corresponding seed pairs in CT and MRI images, and then, transformation was performed using the VariSeed software. The fusion procedures were performed until the positions of the seeds in the CT image corresponded with the positions in the MRI image. The prostate and urethra were contoured manually using MRI images. We contoured the urethra within the prostate gland with the use of a 4-mm circle. We also contoured the urethra outside the prostate gland till the region for which 144 Gy was prescribed.

### Qualitative image analysis of 1.5-T and 3.0-T MRI

Two observers compared 1.5-T MRI and 3.0-T MRI. All images were subjectively scored according to the quality of the following three evaluation items: definition of the prostate outline, urethra identification, and seed identification. A score ranging from 1 to 3 (1 = poor, 2 = moderate, and 3 = good) was assigned to each item. The higher score was regarded as better visualization. We compared the mean scores for each item [2].

### Data analysis

Observer 1 had 10 years of experience in post-implant dosimetry analysis of PPB, and Observer 2 had completed the prostate atlas and contouring modules on www.prostadoodle.com, trained for 1 year in post-implant dosimetry analysis, and had experience in >20 cases.

In the 1.5-T and 3.0-T groups, the interobserver variabilities in DVH parameters, such as prostate D90, prostate V100 (percentage of the prostate volume receiving at least 100% of the prescribed dose), prostate V150 (percentage of the prostate volume receiving at least 150% of the prescribed dose), urethral D5 [dose (Gy) received by 5% of the urethral volume], and urethral V150 (the urethral volume receiving at least 150% of the prescribed dose), were calculated using Pearson’s correlation coefficient (*r*). The interobserver differences in these parameters were analyzed using the paired Student’s *t* test. The scores of imaging quality were estimated using the Mann–Whitney test. A *P*-value < 0.05 was considered to indicate statistical significance. Statistical analysis was performed using IBM SPSS Statistics 22.0.

### Ethical considerations

The study was approved by the ethics review committee of Okayama University Hospital (Approval no. 1801-028; 26 December 2017).

## RESULTS

Baseline characteristics of the 60 patients enrolled are presented in Table 1. There was a significant difference in urethral D5 and urethral V150.

**Table 1.** Patient characteristics

	1.5-T group	3.0-T group	*P*^a^
Intraoperative TRUS prostate volume (cm^3^)	30.5 ± 5.2	30.1 ± 5.4	0.75
Post-implant prostate volume (cm^3^)^b^	29.0 ± 5.1	29.0 ± 5.2	0.97
Implanted seed number	83.3 ± 10.2	83.9 ± 9.6	0.81
Prostate D90 (Gy)^b^	171.0 ± 14.0	172.2 ± 15.6	0.74
Prostate V100 (%)^b^	96.1 ± 2.4	96.8 ± 2.3	0.25
Prostate V150 (%)^b^	64.1 ± 11.5	61.2 ± 12.1	0.33
Urethral D5 (Gy)^b^	244.7 ± 32.2	227.9 ± 32.2	0.047
Urethral V150 (cm^3^)^b^	0.16 ± 0.17	0.08 ± 0.08	0.03

^a^*P*-values were calculated using the unpaired Student’s *t* test and the Mann–Whitney test.

^b^Evaluated by Observer 1.

TRUS = transrectal ultrasound.

**Figure 2.**
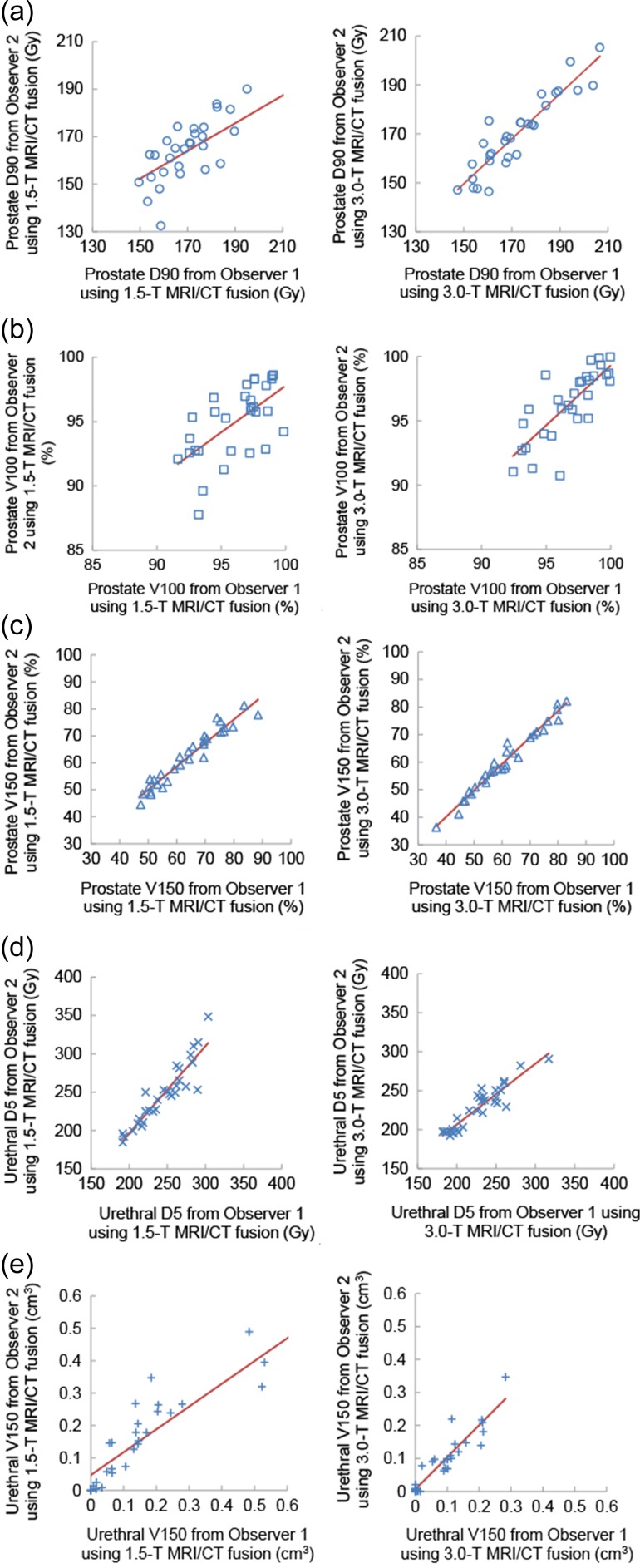
Interobserver variabilities in dose–volume histogram parameters. (a) Prostate D90 (Gy). (b) Prostate V100 (%). (c) Prostate V150 (%). (d) Urethral D5 (Gy). (e) Urethral V150 (ml). MRI, magnetic resonance imaging; CT, computed tomography.

The DVH parameters calculated by Observer 1 and Observer 2 in the 1.5-T and 3.0-T groups are shown in Fig. 2 and Table 2.

**Table 2.** Interobserver variability and differences in DVH parameters

	Observer 1	Observer 2	*r*	*P* (correlation)	*P* (paired *t* test)
1.5-T group					
Prostate D90	171.0 ± 14.0	164.5 ± 12.5	0.65	<0.001	0.004
Prostate V100	96.1 ± 2.4	95.0 ± 2.8	0.62	<0.001	0.011
Prostate V150	64.1 ± 11.5	62.3 ± 10.3	0.97	<0.001	0.002
Urethral D5	244.7 ± 32.2	247.1 ± 39.7	0.92	<0.001	0.417
Urethral V150	0.16 ± 0.17	0.16 ± 0.14	0.88	<0.001	0.988
3.0-T group					
Prostate D90	172.2 ± 15.6	170.1 ± 15.6	0.93	<0.001	0.068
Prostate V100	96.8 ± 2.3	96.3 ± 2.7	0.82	<0.001	0.130
Prostate V150	61.2 ± 12.1	60.5 ± 11.9	0.98	<0.001	0.084
Urethral D5	227.9 ± 32.2	228.4 ± 27.1	0.93	<0.001	0.842
Urethral V150	0.08 ± 0.08	0.09 ± 0.09	0.93	<0.001	0.496

DVH = dose–volume histogram.

Pearson’s correlation coefficients for all DVH parameters were larger in the 3.0-T group than in the 1.5-T group, and, in the 3.0-T group, no significant differences were noted in any of the DVH parameters between Observer 1 and Observer 2 (0.068 ≤ *P* ≤ 0.842). In the 1.5-T group, significant differences were observed for prostate D90, prostate V100 and prostate V150 (*P* < 0.05) between Observer 1 and Observer 2.

Comparisons of the image qualities of 1.5-T MRI and 3.0-T MRI are presented in Table 3. All mean scores of 3.0-T MRI were higher compared with those of 1.5-T MRI, and significant differences were observed for all items, except the prostate outline by Observer 1.

**Table 3.** Median and mean imaging scores of 1.5-T and 3.0-T magnetic resonance imaging for prostate outline, urethra identification, and seed identification

	1.5-T		3.0-T		*P*
	Median	Mean	Median	Mean	
Observer 1					
Prostate outline	3	2.6	3	2.8	0.360
Urethra identification	3	2.6	3	2.9	0.008
Seed identification	2	1.8	3	2.9	<0.001
Observer 2					
Prostate outline	3	2.6	3	2.8	0.025
Urethra identification	2	1.8	2	2.4	0.004
Seed identification	2	1.7	3	2.9	<0.001

## DISCUSSION

CT-based prostate contouring has improved with current techniques; however, it provides comparatively poor prostate delineation when compared with MRI [2]. Initial research showed that CT-based prostate volumes were larger than MRI-based prostate volumes by 24–40% in patients treated with external beam radiation therapy (EBRT) for prostate cancer [17], and this increase is probably associated with the inclusion of anterior and lateral neurovascular bundles or other soft-tissue structures. Additionally, we believe that posterior prostate contouring is inaccurate, particularly at the apex, because the rectum–prostate border is difficult to identify on the CT image.

The contours used for post-implant dosimetry in patients treated with PPB may indicate a higher extent of contouring variability when compared with the findings in patients treated with EBRT, because there is an additional compounding element of prostatic edema [11, 18, 19]. Another issue with prostate contouring in post-implant CT images is the presence of implanted seeds, which introduce metal artifacts and reduce image quality [7]. After PPB, considerable improvements in prostate contouring variability can be achieved with MRI when compared with CT [7, 11]. The most difficult aspect of prostate contouring is the identification of the cranial and caudal extents of the prostate (i.e. base and apex). With clear soft-tissue contrast on performing MRI, which allows the differentiation of the prostate from neighboring structures, the identification of the cranial and caudal extents is improved [7].

The coils with a narrow sensitivity area, such as the endorectal coil, improve the signal-to-noise ratio in the prostate area on MRI [20]. However, if coils of the same sensitivity area are used, the signal-to-noise ratio is greater on 3.0-T MRI than 1.5-T MRI because 3.0-T MRI has a higher magnetic field. The use of 3.0-T MRI could have resulted in improvements in spatial MRI resolution and anatomical details [16]. Our data of image quality scores indicated that 3.0-T MRI was superior to 1.5-T MRI in terms of prostate edge definition and urethral identification. A previous study reported that there was no significant difference in prostate contouring variability after PPB between 1.5-T MRI and 3.0-T MRI; however, the results were limited owing to the use of a cardiac coil in the 1.5-T MRI system and a torso coil in the 3.0-T MRI system, because the sensitivity area of the cardiac coil was narrower than that of the torso coil [7]. In our study, we used a cardiac coil in the 1.5-T MRI system and a body array anterior and posterior coil in the 3.0-T MRI system. It was believed that there was an advantage in the strong magnetic field of 3.0-T MRI, because these coils had an approximately equivalent sensitivity area.

There have been issues with image fusion accuracy in MRI/CT fusion image-based post-implant dosimetry [13, 21, 22]. However, our image quality scores of seed identification in the 3.0-T group were superior to those in the 1.5-T group. We have recognized the implanted seeds as artifacts of black holes, but originally titanium seeds have been less susceptible to artifacts. In 1.5-T MRI, artifacts have been significantly small to recognize seeds clearly, but because 3.0-T MRI artifacts have been increased, it suggested that visibility of the seeds improved in 3.0-T MRI. Therefore, we considered that 3.0-T MRI/CT image fusion by seed matching is more accurate than 1.5-T MRI/CT image fusion.

In our study, there were significant differences in prostate DVH parameters in the 1.5-T group between Observer 1 and Observer 2, but there were no significant differences in the 3.0-T group. Additionally, Pearson’s correlation coefficients for all DVH parameters were larger in the 3.0-T group than in the 1.5-T group. Thus, the interobserver variabilities in prostate DVH parameters in MRI/CT fusion image–based post-implant dosimetry were smaller in the 3.0-T group than in the 1.5-T group, suggesting that 3.0-T MRI is preferable for the MRI/CT fusion image–based post-implant dosimetry used in this study.

The present study has some limitations. This was a retrospective study. Therefore, we could not compare 1.5-T and 3.0-T findings in the same patient. However, with regard to patient characteristics, there was a significant difference only in urethral D5 and urethral V150, and the difference was small. Moreover, we used two MRI machines from two different companies, and the coils used for 1.5-T and 3.0-T MRI systems were different—a cardiac coil was used in the 1.5-T MRI system and a body array anterior and posterior coil was used in the 3.0-T MRI system. In addition, the imaging conditions were different between 1.5-T MRI and 3.0-T MRI. However, these coils had an approximately equivalent sensitivity area. Thus, we considered that the 1.5-T and 3.0-T groups could be compared; and although Observer 2 had fewer years of experience in post-implant dosimetry, we assessed that he was well trained and had sufficient experience to analyze the interobserver variability.

Accurate post-implant dosimetry ensures a successful brachytherapy program because post-implant dosimetry has been shown to be correlated with patient prognosis [23] and toxicity [24]. However, dosimetry depends on the precision of contouring, and a small difference in contours can lead to actual changes in dosimetry [18]. Our findings suggested that the interobserver variability in prostate contouring in the MRI/CT fusion image–based post-implant dosimetry analysis of brachytherapy was lower with 3.0-T MRI than with 1.5-T MRI in this study.

## CONCLUSION

We found that the interobserver variability in prostate contouring in the MRI/CT fusion image–based post-implant dosimetry analysis of brachytherapy was lower with 3.0-T MRI than with 1.5-T MRI in this study. Thus, it is suggested that 3.0-T MRI is the preferred modality for MRI/CT fusion-based postimplant dosimetry.

## CONFLICT OF INTEREST

The authors declare that there are no conflicts of interest.

## FUNDING

None declared.
